# Inhibition of NADPH Oxidase Activation in Oligodendrocytes Reduces Cytotoxicity Following Trauma

**DOI:** 10.1371/journal.pone.0080975

**Published:** 2013-11-19

**Authors:** Joshua T. Johnstone, Paul D. Morton, Arumugam R. Jayakumar, Andrea L. Johnstone, Han Gao, Valerie Bracchi-Ricard, Damien D. Pearse, Michael D. Norenberg, John R. Bethea

**Affiliations:** 1 The Miami Project to Cure Paralysis, University of Miami, Miami, Florida, United States of America; 2 Department of Pathology, Miller School of Medicine, University of Miami, Miami, Florida, United States of America; 3 Department of Neurosurgery, Miller School of Medicine, University of Miami, Miami, Florida, United States of America; 4 Department of Biochemistry and Molecular Biology, University of Miami, Miami, Florida, United States of America; 5 South Florida Foundation for Research & Education Inc, Veterans Affairs Medical Center, Miami, Florida, United States of America; 6 Department of Microbiology and Immunology, Miller School of Medicine, University of Miami, Miami, Florida, United States of America; Hertie Institute for Clinical Brain Research, University of Tuebingen, Germany

## Abstract

Spinal cord injury is a debilitating neurological disorder that initiates a cascade of cellular events that result in a period of secondary damage that can last for months after the initial trauma. The ensuing outcome of these prolonged cellular perturbations is the induction of neuronal and glial cell death through excitotoxic mechanisms and subsequent free radical production. We have previously shown that astrocytes can directly induce oligodendrocyte death following trauma, but the mechanisms regulating this process within the oligodendrocyte remain unclear. Here we provide evidence demonstrating that astrocytes directly regulate oligodendrocyte death after trauma by inducing activation of NADPH oxidase within oligodendrocytes. Spinal cord injury resulted in a significant increase in oxidative damage which correlated with elevated expression of the gp91 phox subunit of the NADPH oxidase enzyme. Immunohistochemical analysis confirmed the presence of gp91 phox in oligodendrocytes *in vitro* and at 1 week following spinal cord injury. Exposure of oligodendrocytes to media from injured astrocytes resulted in an increase in oligodendrocyte NADPH oxidase activity. Inhibition of NADPH oxidase activation was sufficient to attenuate oligodendrocyte death *in vitro* and at 1 week following spinal cord injury, suggesting that excitotoxicity of oligodendrocytes after trauma is dependent on the intrinsic activation of the NADPH oxidase enzyme. Acute administration of the NADPH oxidase inhibitor apocynin and the alpha-amino-3-hydroxy-5-methylisoxazole-4-propionate channel blocker 2,3-dihydroxy-6-nitro-7-sulfamoyl-benzo[f]quinoxaline-2,3-dione significantly improved locomotor behavior and preserved descending axon fibers following spinal cord injury. These studies lead to a better understanding of oligodendrocyte death after trauma and identify potential therapeutic targets in disorders involving demyelination and oligodendrocyte death.

## Introduction

Oxidative stress and reactive oxygen species (ROS) production play a predominant role in the secondary injury mechanisms of spinal cord injury (SCI) [1]. Under normal conditions, ROS are produced as a byproduct of cellular respiration, and are important regulators of numerous intracellular signaling pathways that control cell adhesion, migration, differentiation, proliferation, and gene expression [2-4]. Although there are many enzymes that produce ROS, recent studies have demonstrated that under conditions of glutamate excitotoxicity, oxidative stress and free radical production are mediated by increased nicotinamide adenine dinucleotide phosphate (NADPH) oxidase activity [5]. Cortical neurons treated with the excitotoxin N-methyl-D-aspartate (NMDA) exhibited a rapid increase in intracellular superoxide accumulation that correlated with increased neuronal death. Treatment with the NADPH oxidase inhibitor apocynin, or genetic deletion of the p47 phox subunit of the NADPH oxidase complex, was sufficient to block NMDA-stimulated superoxide production and attenuate neuronal death [5]. These studies identified NADPH oxidase as a key downstream regulator of glutamate receptor-mediated excitotoxic cell death mechanisms. 

Oligodendrocytes are particularly susceptible to oxidative stress as a result of their poor antioxidant defense system [6-11]. The production of ROS is ameliorated in other cell types by glutathione, a potent antioxidant tripeptide [12]. Astrocytes, which are relatively resistant to oxidative stress, produce approximately 5 mM glutathione [6]. Oligodendrocytes have less than 1 mM glutathione, making them 5 times more vulnerable to oxidative stress than astrocytes. In addition to their relatively poor antioxidant production, oligodendrocytes utilize a large amount of metal ions as cofactors in many biological processes [13,14]. Intracellular accumulation of iron can stimulate the conversion of hydrogen peroxide to a hydroxyl radical, resulting in an increase in oxidative stress [6,15]. Since oligodendrocytes lack the appropriate antioxidant defense, their fate in the presence of free radical insult is largely dependent upon antioxidant support from other cells. 

Previous work by our laboratory demonstrated that astrocytes can directly influence glutamate-mediated oligodendrocyte toxicity after injury through activation of the astroglial nuclear factor – κB (NF – κB) transcription factor [16]. Using a transgenic mouse expressing a dominant negative form of the inhibitor of κBα (IκBα) protein under the control of the glial fibrillary acidic protein (GFAP) promoter (GFAP-IκBα-dn), we showed that prevention of NF-κB activation in astrocytes significantly improved hind limb locomotor recovery and preserved myelin and axon tracts following a moderate contusion SCI [17,18] Furthermore, suppression of astroglial NF – κB activation after trauma resulted in a reduction in oligodendrocyte death by preventing zinc uptake by astrocytes [16]. These experiments showed that preservation of extracellular zinc concentrations was sufficient to inhibit glutamate-mediated excitotoxicity, but the signaling mechanisms within oligodendrocytes which mediated the cell death response were not determined. In the present study, we sought to examine mechanisms intrinsic to oligodendrocytes which contribute to their toxicity following SCI. By elucidating these signaling mechanisms, we hope to illuminate pathways that can be targeted for therapeutic strategies to improve oligodendrocyte survival and induce functional recovery after SCI. 

## Methods

### Mice

Female mice expressing a dominant negative form of the IκBα protein in GFAP expressing cells (GFAP-IκBα-dn) were produced and characterized as previously described [16-20]. Wild-type littermates served as controls. *Cybb* ^*-/-*^ mice lacking gp91 phox (B6.129S6-*Cybbtm1Din*/J) were obtained from the Jackson Laboratory (Bar Harbor, ME). Mice were housed in a 12 h light/dark cycle in a virus/antigen free environment. All mice had free access to food and water throughout the course of the study.

### Spinal Cord Injury

All surgical procedures were performed in the Surgical Core Facility of The Miami Project to Cure Paralysis, according to the protocols and guidelines approved by the Institutional Animal Care and Use Committee (IACUC) of the University of Miami. The studies shown here were approved by the University of Miami IACUC. Adult female mice 3-5 months of age (20-24g in weight) underwent a moderate to severe contusion injury to the T9 segment of spinal cord using the Electromagnetic Spinal Cord Injury Device (Ohio State University). In brief, mice received a laminectomy between T8 and T10 vertebral segments. Mice were injured with a spinal cord displacement of 0.5 - 0.6 cm. Following injury, mice were treated with topical antibiotics and subcutaneous Ringer’s solution to reduce fluid loss. Daily administration of Ringer’s solution to prevent dehydration and gentamicin to prevent urinary tract infections was given for the 5 days after injury or as needed. Bladder expressions were performed twice daily until the reacquisition of bladder control.

### Astrocyte Cultures

Astrocyte cultures were prepared as previously described [16,21]. Primary cortical astrocytes were isolated from one to two day old wild-type (WT) and GFAP-IκBα-dn mice. Following removal of the meninges, the cortex was minced, dissociated, passed through sterile nylon sieves, and plated in 30 mm plates with Dulbecco’s modified Eagle medium (DMEM; Life Technologies, Gaithersburg, MD) containing penicillin, streptomycin, and fetal bovine serum. Cultures were incubated at 37°C in a humidified incubator with 5% CO_2_ for 10 days, at which time the fetal bovine serum was replaced with horse serum. After 14 days, astrocyte cultures were treated with dibutyryl cyclic adenosine monophosphate (Sigma, St. Louis, MO) to enhance cell proliferation and maturation [22]. Studies were performed when cultures were 3 - 4 weeks old. 

### *in vitro* Injury Model

The *in vitro* trauma was induced using a fluid percussion device developed by Sullivan et al. [23] and modified for cell culture [24]. Astrocyte cultures were filled with astrocyte media and wrapped airtight in cellophane. The culture plates were placed in the cell chamber and the chamber was filled with saline to create a fluid system. Five atmospheres of pressure was applied twice to the cultures for 25 millisecond durations. Pressure was monitored with a PowerLab system (ADInstruments, Inc., Colorado Springs, CO) with a high-speed pressure transducer. 

### Oligodendrocyte Isolation

The oligodendrocyte isolation procedure was modified from an existing protocol [25], and was performed as previously described [16]. Spinal cords from 2 -3 month old female mice were removed and dissociated using a buffer consisting of Hanks’ Balanced Salt Solution (HBSS, Gibco) containing 10 U/ml papain (Sigma), 5.5 mM cysteine (Sigma), 2.5 mM ethylenediaminetetraacetic acid (EDTA; Sigma), 20 mM 4-(2-hydroxyethyl)-1-piperazineethanesulfonic acid (HEPES; Sigma), 0.01 N sodium hydroxide (NaOH). Dissociation of the spinal cords was stopped by adding HBSS containing 250 U/ml deoxyribonuclease I (DNase I; Sigma), 0.2% bovine serum albumin (BSA; Sigma), 10 μg/ml gentamicin (Gibco), and 20 mM HEPES (Sigma), pH 7.4 for 15 minutes at 37°C. The cell suspension was filtered through a 100 micron filter (BD Biosciences) followed by a 40 micron filter, and centrifuged at 1500 rpm for 5 minutes and resuspended in a 10% Percoll (GE Healthcare) solution. The 10% Percoll suspension was added to a discontinuous 15/60% Percoll gradient in an Oak Ridge centrifugation tube (Nalgene) and centrifuged at 30,000 *g* for 30 minutes at 4°C. The 15/60% interface was removed, washed, centrifuged and resuspended in DMEM containing 10% fetal bovine serum (ThermoScientific) and 10 μg/ml gentamicin. Cells were plated for two days, at which time the media was changed to 30% B104 conditioned media (CM) and 70% serum-free media (SFM), containing DMEM with 25 μg/ml transferrin (Sigma), 30 nM triiodothyronine (T3; Calbiochem), 20 nM hyrdrocortisone (Sigma), 20 nM progesterone (Sigma), 10 nM biotin (Sigma), 1X Trace Element Mix B (Mediatech), 30 nM selenium (Sigma), 5 μg/ml insulin (Sigma), 1 μg/ml putrescine (Sigma), 0.1% BSA (Sigma), 10 μg/ml gentamicin. To produce mature oligodendrocytes, cultures were plated in SFM alone for 7-10 days. The resulting cultures contained oligodendrocytes with large flat processes, and labeled with CC 1, O4, O1, and GalC.

### Cytotoxicity Assay

Oligodendrocyte cytotoxicity was assessed using the trypan blue exclusion method, as previously described [16,26]. The media was collected and oligodendrocytes were trypsinized with 0.05% trypsin-EDTA (Gibco) and resuspended in phosphate buffered saline (PBS). Cells were stained with 0.4% Trypan blue dye and quantified using a hemacytometer. 

### NADPH Oxidase Activity Assay

NADPH oxidase activity was determined using chemiluminescence to detect superoxide production as previously described [27,28]. At 3 hrs post-treatment with injured astrocyte media, oligodendrocytes were collected in Kreb’s HEPES buffer consisting of 99 mM NaCl, 4.69 mM KCl, 1.87 mM CaCl_2_, 1.2 mM KH_2_PO_4_, 25 mM NaHCO_3_, 20 mM Na-Hepes, 11.1 mM Glucose, pH = 7.4. Samples were centrifuged at 2,000 rpm and resuspended in Kreb’s HEPES buffer. A mixture of 50 μM Lucigenin and 1 mM of NADPH was added to each sample and luminescence was measured using a luminometer (Turner Designs, TD-20/20). 

### Terminal Deoxynucleotidyltransferase (TdT)-mediated dUTP-Biotin Nick End Labeling (TUNEL) Stain

Cell death was assessed using the Apoptag® Plus Fluorescein In Situ Apoptosis Detection Kit (Millipore) according to the manufacturer’s instructions. 

### Immunohistochemistry

Mice were perfused with 4% paraformaldehyde, and a 1 cm segment (from 5mm rostral to 5mm caudal of the lesion epicenter) of the spinal cord was removed and post-fixed in 4% paraformaldehyde overnight. Samples were incubated in 25% sucrose and cut into 20 or 25 µm thick sections. Sections were blocked (5% normal goat serum in .4% Triton-X100 in PBS) for 1 hr at room temperature and incubated at 4°C overnight in primary antibodies [glutathione-S-transferase π (GSTπ) (Millipore; 1:2000), neurofilament-H (Covnace; 1:500), microtubule-associated protein 2 (MAP2; Sigma; 1:500), serotonin (5-hydroxytryptamine; 5-HT; Immunostar; 1:2000)]. Slides were treated with species-specific fluorescent secondary antibodies (Alexa Fluor 488 and Alexa Fluor 594, 1:500; Molecular Probes, Eugene, OR) and mounted using DAPI mounting medium (Vectashield, Vector Laboratories, Burlingame, CA). Sections lacking primary antibody treatment were used as negative controls.

### Stereology

Serial spinal cord sections spaced 500 µM apart were counted using Stereo Investigator (MBF Biosciences) software with a 63x objective under blind conditions. For each section, a 40 x 40 µM counting frame was used to count cells at 200 µM intervals. 

### Western Blot

A 1 cm segment of spinal cord centered on the lesion site was homogenized in lysis buffer (1 M Tris-HCl, pH=7.4, 5 M NaCl, 1% Triton X-100, 0.5M EDTA) supplemented with Complete protease inhibitor (Roche). Protein concentrations were determined by Lowry assay using the *Dc* Protein Assay kit (BioRad; Hercules, CA). Equal amounts of protein (30 µg) was separated by SDS-PAGE and transferred to a nitrocellulose membrane (BioRad). The blots probed with primary antibody [gp91 phox (BD Transduction Laboratories; 1:100) β-tubulin (Sigma-Aldrich; 1:500)] in blocking solution overnight at 4°C, and for 1 hr at room temperature with a species-specific horseradish peroxidase (HRP)-conjugated secondary antibody (1:2000, Amersham Biosciences, NJ, USA). Protein densitometry analysis was performed using Quantity One (BioRad) software.

### Oxyblot Slot Blot

The Oxyblot was conducted using the Oxyblot Oxidizied Protein Assay Kit (Chemicon International) according to manufacturer’s specifications. 

### *In Vivo* Treatments

Apocynin was dissolved in 1% EtOH and saline. Mice received intraperitoneal injections of vehicle (1% EtOH and saline) or 10 mg/kg apocynin 1 hr after SCI. Mice received injections of 10 mg/kg apocynin or vehicle daily for 1 week. 

### Combined Treatments

NBQX and apocynin were dissolved in water and apocynin was heated to 65°C until completely in solution. Mice received intraperitoneal injections of vehicle (water), 30 mg/kg NBQX, 10 mg/kg apocynin, or NBQX + apocynin 1 hr after SCI, and continued to receive daily doses for 1 week.

### Behavioral Assessment

Mice were placed in an open field for 4 minutes, and their hind limb locomotor activity was scored according to the Basso Mouse Scale [29]. Behavioral scoring was conducted under blind conditions by two observers. Additional deficits in motor control were assessed by grid-walk analysis pre-injury (baseline) and at 3, and 6 weeks post-SCI by grid-walk analysis. Mice crossed a 1-m-long path with random assigned spacing (0.5–5 cm), as described previously [30]. The number of footfalls and total number of steps taken were recorded, and the stepping efficiency was calculated by: (number of footfalls-total number of steps)/total number of steps.

### Histological Analysis

Sets of cross-sections at a spacing of 500 µm and ranging from 3.5 mm rostral to 3.5 mm caudal of the lesion epicenter were stained with Luxol Fast Blue and analyzed using Stereo Investigator (MBF Biosciences) software. Intact white matter and the lesion site were contoured on each section, and the total volume was calculated using stereological principles.

### Serotonin Quantification

Spinal cord sections 3 mm caudal to the lesion site were imaged using confocal microscopy, and the relative fluorescence intensity was quantified by ImageJ software. The maximum background threshold was determined for each image and set as the minimum threshold for intensity quantification.

### Statistics

Statistical assessment was performed using one or two-way ANOVA, followed by appropriate post-tests. Student’s T test was used for single comparisons. Chronic behavioral evaluations were calculated using the Kruskal-Wallis significance test to assess if the treatments resulted in an overall significant change from controls. P values equal to or less than 0.05 will be considered statistically significant. Error bars indicate the standard error of the mean. 

## Results

### Astroglial NF-κB activation contributes to the oxidative stress response 1 week after SCI

**Figure 1:**
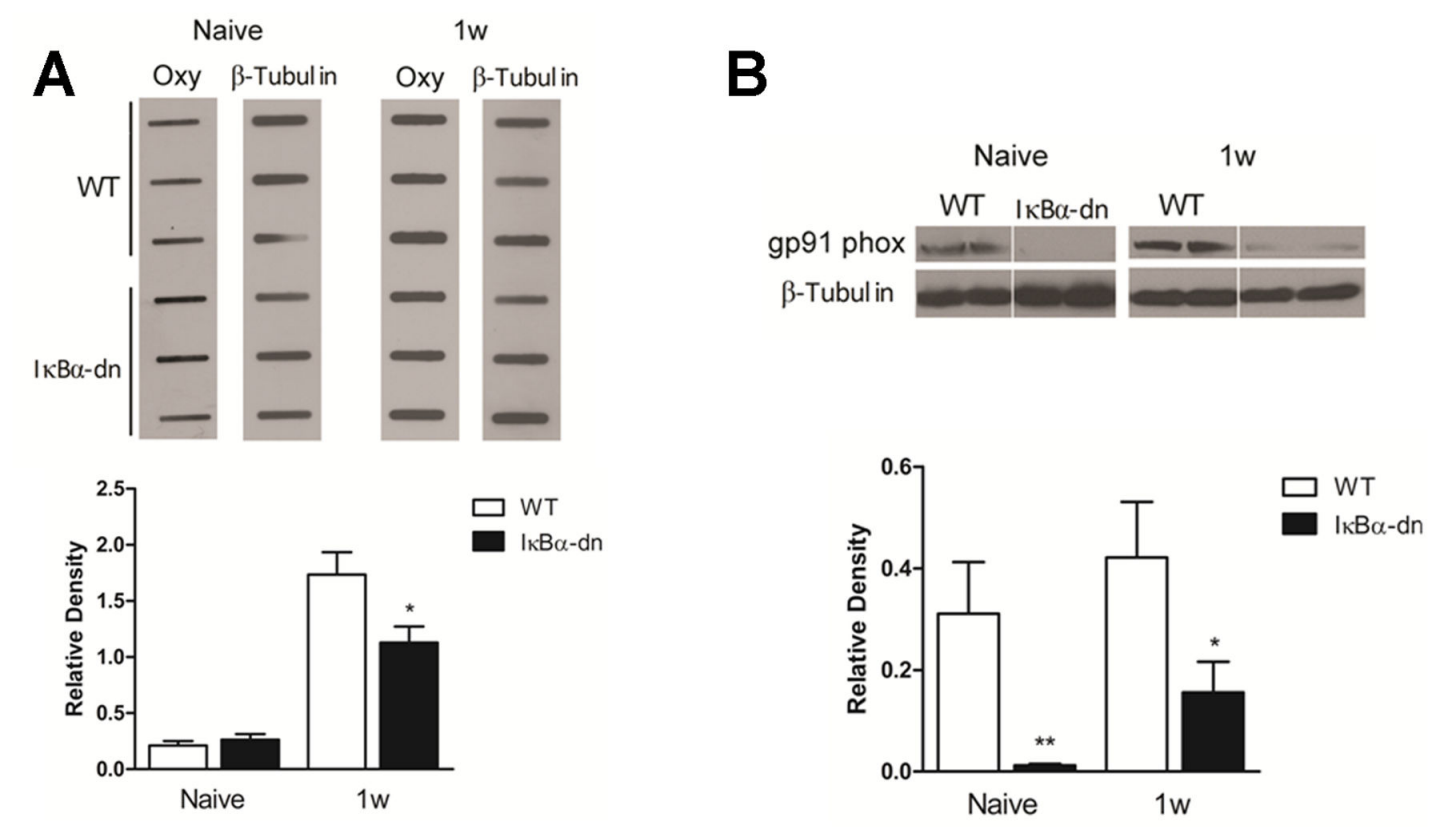
Astroglial NF-κB inactivation reduces oxidative damage and gp91 phox expression. 1 week following SCI. (A) Oxyblot analysis showed reduced levels of oxidatively damaged proteins in IκBα-dn spinal cords 1 week after injury (n=7-9). Results are normalized to β-tubulin levels and expressed as relative density. *, p < 0.05 compared to 1w WT samples. (B) Western blot analysis of gp91 phox levels in naïve WT and IκBα-dn and at 1 week following SCI (n = 7-13). Results are normalized to β-tubulin levels and expressed as relative density. *, p ≤ 0.05 compared to naïve WT samples; **, p ≤ 0.01 compared to 1w WT. Error bars indicate the standard error of the mean.

Previously we determined that mice lacking functional NF-κB in astrocytes possessed a significant reduction in the amount of oligodendrocyte death at 1 week following a moderate contusion SCI [16]. We determined that astrocytes can directly induce oligodendrocyte cytotoxicity after injury through glutamate excitotoxicity [16]. Since glutamate excitotoxicity is tightly coupled to oxidative stress, we wanted to determine if reducing NF-κB activation in astrocytes attenuates ROS-mediated damage following SCI. To quantify the level of protein oxidation in wild-type (WT) mice and mice lacking functional NF-κB activation in astrocytes (GFAP-IκBα-dn), protein lysates isolated from spinal cords 1 week after a moderate contusion SCI were subjected to Oxyblot analysis (Figure 1A). In naïve animals, both WT and GFAP-IκBα-dn mice displayed relatively low levels of basal oxidative damage [0.2105 ± 0.04 and 0.2637 ± 0.05 Relative Density (R.D.)]. At one week after SCI, oxidative damage in WT and GFAP-IκBα-dn mice was significantly elevated from the corresponding controls. When compared to the injured WT condition (1.735 ± 0.2 R.D.), GFAP-IκBα-dn mice (1.128 ± .14 R.D.) exhibited significantly less oxidative damage, thereby providing evidence that the NF-κB transcription factor in astrocytes is a regulator of the oxidative stress response after injury. 

 NADPH oxidase activation has been identified as a key mediator of free radical production and subsequent cell death [5,31]. To determine if reduced levels of oxidative damage in GFAP-IκBα-dn animals after SCI could be the result of reduced NADPH oxidase expression after SCI, naïve and injured WT and GFAP-IκBα-dn spinal cords were subjected to Western blot analysis for the gp91 phox subunit of the NADPH oxidase enzyme (Figure 1B). In naïve mice, WT spinal cords (0.3105 ± 0.101 R.D.) contained significantly higher levels of gp91 phox than GFAP-IκBα-dn animals (0.0124 ± 0.003 R.D.). By one week after SCI, WT gp91 phox levels (0.4213 ± 0.109 R.D.) remained significantly elevated when compared to GFAP-IκBα-dn animals (0.1559 ± 0.061 R.D.). The reduction in gp91 phox expression in GFAP-IκBα-dn mice correlates with the previously documented reduction in oxidative damage and oligodendrocyte death in these animals at 1 week after SCI [16].

### Oligodendrocytes express NADPH oxidase

Excessive AMPA receptor activation on oligodendrocytes induces cell death through excitotoxic mechanisms and subsequent ROS production [5,32,33]. However, the mechanisms within oligodendrocytes that regulate excitotoxic cell death and ROS production remain unclear. To date, studies investigating the role of NADPH oxidase within the nervous system have focused primarily on microglia, neurons, astrocytes, endothelial cells, and infiltrating leukocytes [5,32,33]. There are few accounts of NADPH oxidase expression and function within oligodendrocytes [34]. Since oligodendrocytes are known to be susceptible to glutamate excitotoxicity, we investigated whether oligodendrocytes express components of the NADPH oxidase enzyme and whether activation of this enzyme is sufficient to trigger oligodendrocyte death.

**Figure 2:**
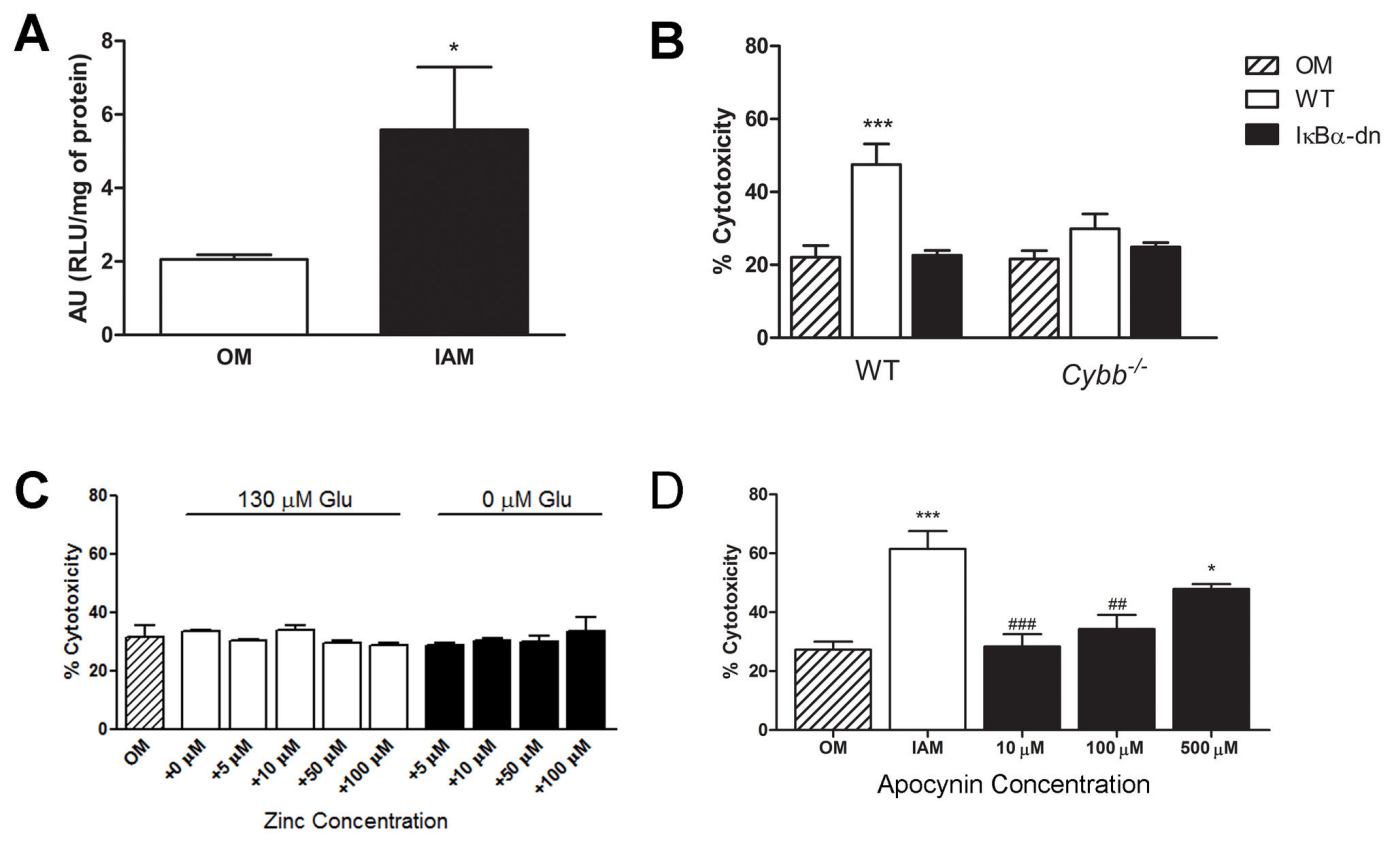
NADPH oxidase induction in oligodendrocytes increases cytotoxicity. (A) Injured astrocytes stimulate NADPH oxidase activity in oligodendrocytes. NADPH oxidase activity was measured in cultures treated with either oligodendrocyte media alone (OM) or injured astrocyte media (IAM) for 3 hrs. Results are expressed as arbitrary units (AU = relative light units (RLU) per mg of protein. (n = 6). *, p ≤ 0.05. (B) Genetic deletion of gp91 phox reduces astrocyte-mediated oligodendrocyte death. Oligodendrocytes from WT or Cybb^-/-^ mice were treated with 3 hr post-injury WT or IκBα-dn astrocyte media (n=3). ***, p ≤ 0.001 relative to WT OM. (C) Oligodendrocytes lacking NADPH oxidase are immune to glutamate excitotoxicity. Oligodendrocytes isolated from Cybb-/- mice were treated with 130 µM glutamate in the presence of varying levels of zinc (n=3). (D) Pharmacological inhibition of NADPH oxidase reduces oligodendrocyte death following treatment with injured astrocyte media *in* *vitro*. Oligodendrocytes were pre-treated with varying concentrations of apocynin for 30 min prior to a 3 hr treatment with injured astrocyte media (n = 4). OM = oligodendrocyte media. IAM = 3 hr post-injury WT astrocyte media. *, p ≤ 0.05 compared to OM; ***, p ≤ 0.001 compared to OM; ##, p < 0.01 compared to IAM; ###, p < 0.001 compared to IAM. Error bars indicate the standard error of the mean.

To further confirm the function of the NADPH oxidase enzyme within oligodendrocytes after injury, we treated oligodendrocyte cultures with media from injured astrocyte cultures known to induce glutamate excitotoxic oligodendrocyte death [16] and performed a chemiluminescent NADPH oxidase activity assay (Figure 2A). Oligodendrocytes exposed to control oligodendrocyte media showed low levels of basal NADPH oxidase activity [2.06 ± .13 arbitrary units (AU)], confirming a low level of NADPH oxidase activity under normal conditions. However, following exposure to injured astrocyte media, NADPH oxidase activity was significantly elevated in oligodendrocyte cultures (5.59 ± 1.71 AU), not only providing confirmatory data that oligodendrocytes express NADPH oxidase but also providing evidence that injured astrocytes are capable of activating the NADPH oxidase complex within oligodendrocytes. 

### Genetic deletion of *Cybb* reduces astrocyte mediated oligodendrocyte death

To determine if NADPH oxidase activation within oligodendrocyte is necessary for glutamate excitotoxicity induced by injured astrocytes [16], oligodendrocytes isolated from *Cybb* ^*-/-*^ mice, which are deficient in the gp91 phox subunit of NADPH oxidase, were treated with injured WT or GFAP-IκBα-dn astrocyte media. Cytotoxicity was assessed using a trypan blue exclusion assay in which dying cells will be permeable to the dye (Figure 2B). Injured WT astrocyte media induced a significant increase in WT oligodendrocyte death (22.1 ± 3.25%) over control (47.5 ± 5.67%) and injured GFAP-IκBα-dn media (22.7 ± 1.35%). When oligodendrocytes isolated from *Cybb* ^*-/-*^ animals were treated with injured WT astrocyte media, there was no change in the level of cytotoxicity (29.9 ± 4.04%) from controls, suggesting that injured astrocyte-mediated oligodendrocyte death is dependent upon NADPH oxidase activation within oligodendrocytes.

Previous studies have shown that injured astrocyte-mediated oligodendrocyte death is the result of a reduction in extracellular zinc levels in the presence of high glutamate concentrations. To confirm that glutamate/zinc-mediated oligodendrocyte death is the result of the expression and activation of NADPH oxidase within oligodendrocytes, we treated oligodendrocytes from Cybb^-/-^ mice with elevated extracellular glutamate concentrations in the presence of varying levels of zinc (Figure 2C). Treatment of oligodendrocytes lacking gp91 phox with 130 µM glutamate resulted in no change in cell death over controls regardless of the extracellular zinc concentration, indicating that glutamate/zinc mediated excitotoxicity is mediated by NADPH oxidase within the oligodendrocyte.

### Apocynin reduces oligodendrocyte death *in vitro* and *in vivo* following trauma

To confirm that NADPH oxidase inhibition could be a potential therapeutic target following trauma, oligodendrocytes were pre-treated with apocynin (10 µM, 100 µM, or 500 µM) for 30 min followed by treatment with injured astrocyte media for 3 hrs (Figure 2D). Apocynin is an NADPH oxidase inhibitor and has been reported to block formation of the active enzyme, thereby preventing the production of superoxide free radicals [35]. Following treatment with injured astrocyte media, the levels of cell death significantly increased, as previously shown (61.5 ± 6.02%). However, oligodendrocytes treated with apocynin (10 and 100 µM) were significantly protected from cytotoxicity (10 µM = 28.3 ± 4.2%; 100 µM = 34.3 ± 4.8%). At higher concentrations, apocynin failed to inhibit oligodendrocyte death. This result is supported by findings in the literature showing that at high concentrations apocynin can be toxic [35,36]. 

**Figure 3:**
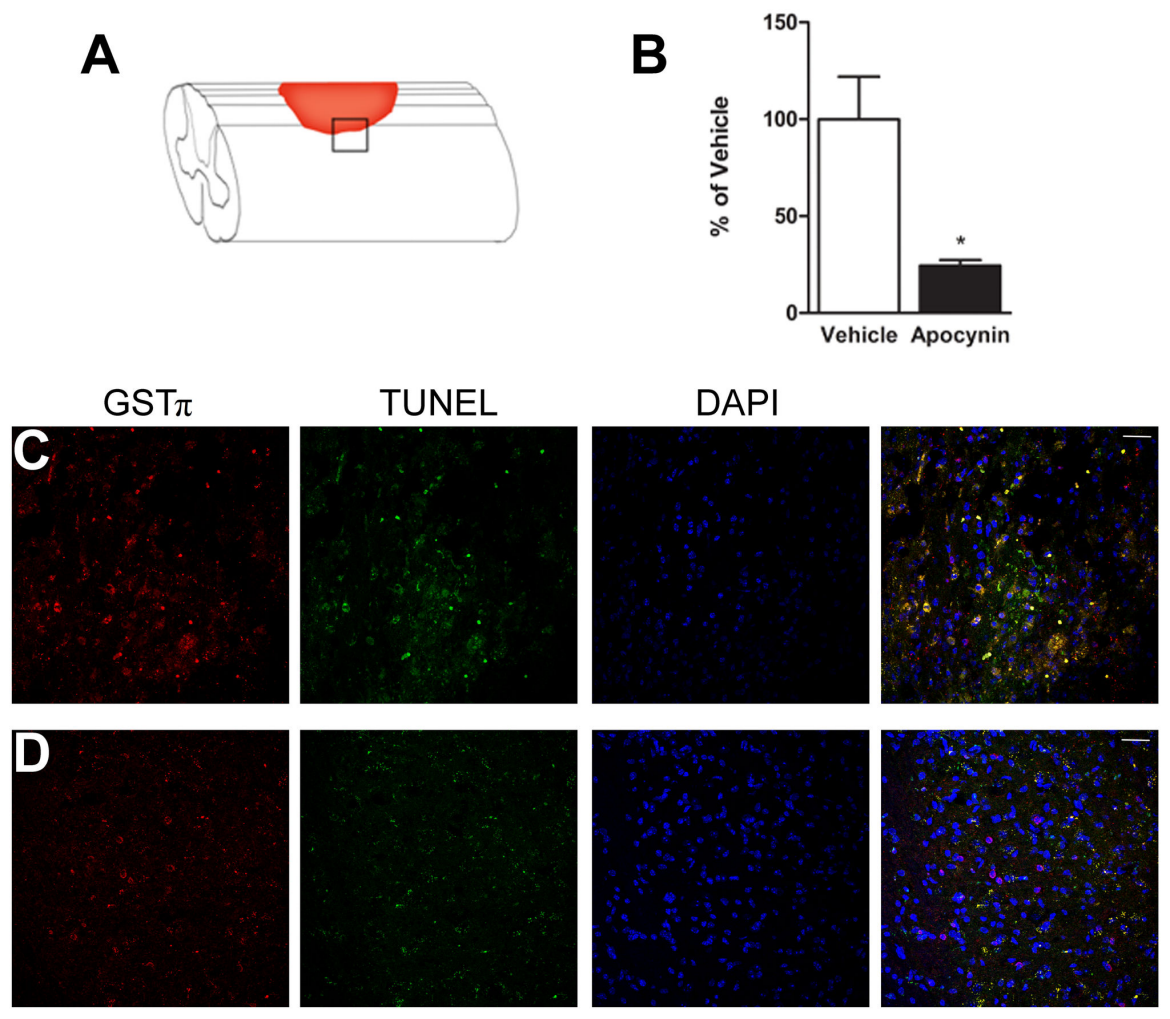
Pharmacological inhibition of NADPH oxidase reduces oligodendrocyte death 1 week after SCI. (A) Diagram showing the location of the lesion site (red area) and location of the images (box). (B) Stereological quantification of the number of GSTπ/TUNEL positive cells in vehicle treated controls and apocynin treated animals (10 mg/kg) revealed a 75% reduction in oligodendrocyte death following apocynin treatment (n = 4). Results are normalized to vehicle. *, p<0.05. (C) Vehicle-treated animals showed many cells double-labeled for the nuclear oligodendrocyte marker GSTπ and TUNEL. (D) Treatment with 10 mg/kg of apocynin 1 hr following SCI and daily for 1 week reduced the number of TUNEL positive cells. Scale bars = 50 µM. Error bars indicate the standard error of the mean.

To determine if apocynin treatment is capable of reducing oligodendrocyte death following SCI, WT mice were subjected to a moderate spinal cord contusion at the thoracic level T9. One hour after injury, mice received a 10 mg/kg intraperitoneal injection of apocynin, and continued to receive daily treatments with apocynin for one week. In other *in vivo* models of neurological disorders using apocynin, dosages ranging from 4 mg/kg to 300 mg/kg were shown to have significant improvements on behavior and survival [37-40]. In our study we chose 10 mg/kg because it is the lowest dosage that yielded the maximal beneficial effect in previous studies. At 1 week following SCI, spinal cords were removed and immunostained with the nuclear oligodendrocyte marker GSTπ and the cell death marker TUNEL. Cells labeled with both markers were quantified using stereological principles to determine the number of oligodendrocytes undergoing cell death. Treatment with apocynin reduced the number of oligodendrocytes (353.7 ± 43.3) that were positive for both TUNEL and GSTπ markers by 75% when compared to vehicle-treated mice (1,451 ± 318) (Figure 3). These results confirm that inhibition of NADPH oxidase after SCI is sufficient to attenuate oligodendrocyte death. 

### Combined inhibition of AMPA receptors and NADPH oxidase results in hind limb locomotor recovery following SCI

**Figure 4:**
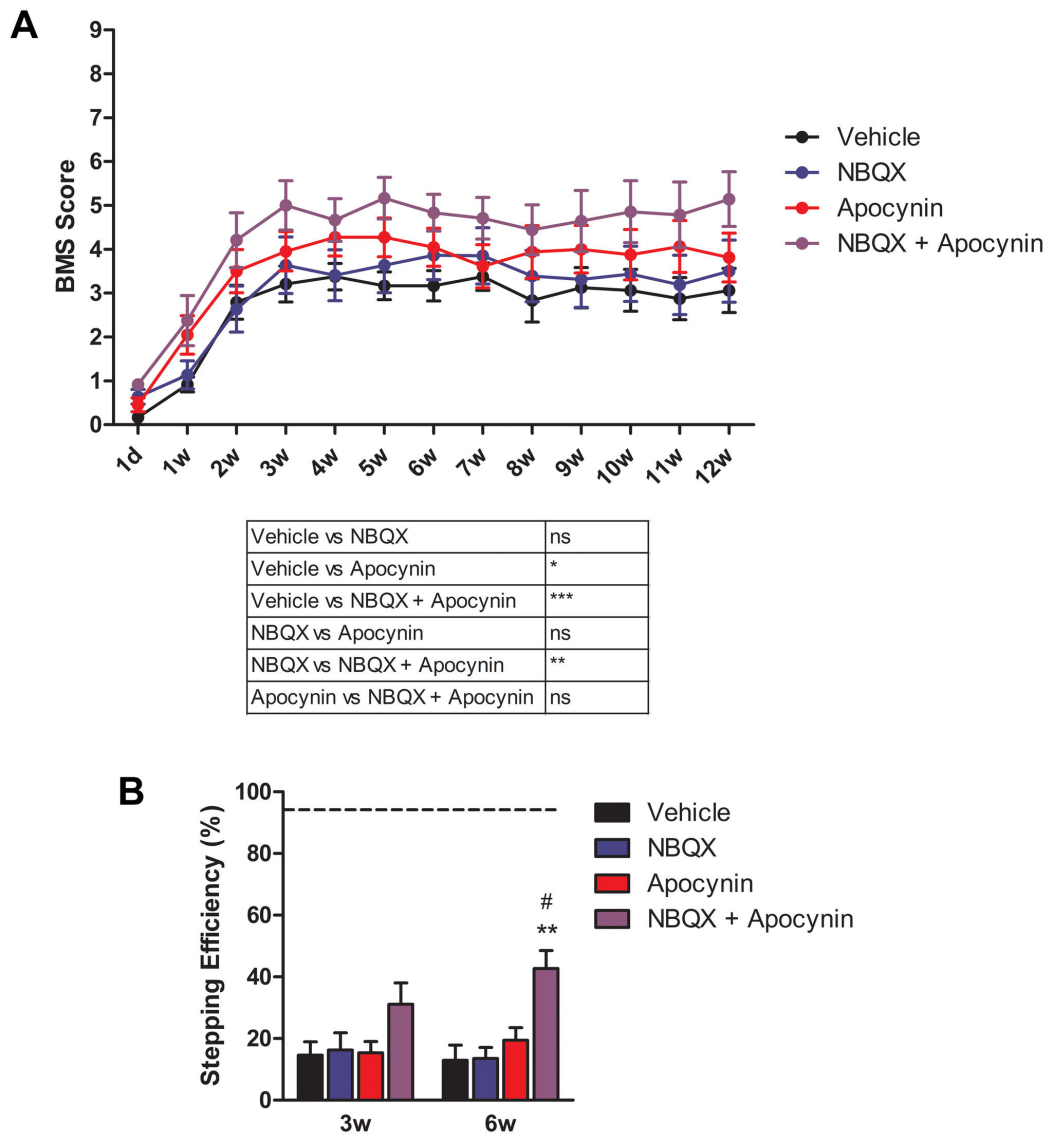
Combined therapy of apocynin and NBQX significantly increases hind limb motor recovery after SCI. Animals received treatment [vehicle (water), NBQX (30 mg/kg), apocynin (10 mg/kg), or NBQX + apocynin)] 1 hr after injury and daily for 1 week after trauma. (A) Assessment of hind limb motor recovery by BMS scoring under blind conditions from 1 day (1d) to 12 weeks (12w) post-injury (n = 11-12). *, p ≤ 0.05; **, p ≤ 0.01; ***, p ≤ 0.001 compared to vehicle. (B) Assessment of hind limb coordination by grid walk analysis (n = 7-12). Results are expressed as stepping efficiency determined by the percent of successful foot placements divided by the total number of steps. Baseline results are depicted as a dashed line. **, p ≤ 0.01 compared to vehicle and NBQX; #, p ≤ 0.05 compared to apocynin. Error bars indicate the standard error of the mean.

Several reports have shown that axonal AMPA receptor activation is necessary for axon dysfunction and degeneration [41,42]. Studies using pharmacological inhibitors of the AMPA receptor, such as NBQX, after SCI have yielded promising results in improving behavioral recovery and preventing tissue damage [43-45]. Based on our previous findings using apocynin to treat SCI, and recognizing that oligodendrocyte preservation alone is likely insufficient to promote full functional recovery after contusive SCI, we employed a combinatorial treatment strategy to reduce oligodendrocyte death and block axonal damage. In order to assess whether targeting axon damage in combination with preventing NADPH oxidase-mediated oligodendrocyte death is a viable combinatorial treatment strategy, mice were treated with either vehicle (water), NBQX (30 mg/kg), apocynin (10 mg/kg), or NBQX + apocynin 1 hr after SCI and daily thereafter for 1 week. Changes in motor function were evaluated weekly for 12 weeks using the BMS hind limb locomotor test (Figure 4A) and at 3 and 6 weeks using the grid walk test (Figure 4B). By one week after SCI, mice began regaining locomotor recovery. At 12 weeks post-SCI, treatment with vehicle or NBQX alone resulted in a BMS hind limb motor score equivalent to plantar placement of the hind paw without weight support or stepping (BMS = 3). NBQX alone did not result in any significant improvement in recovery when compared to vehicle animals. Apocynin treatment induced occasional hind limb stepping (stepping less than 50% of the time moving forward; BMS = 4) which was a slight, but overall significant, improvement in hind limb motor function when compared to vehicle treated animals. Combined treatment of NBQX and apocynin induced frequent stepping recovery with some coordination (BMS = 5 and 6) which resulted in a significant recovery of locomotor behavior. Grid walk analysis further demonstrated the combination treatment group significantly improved stepping efficiency at 6 weeks after injury (42.7 ± 5.89%), an effect lacking in the single treatment groups (Vehicle: 13.0 ± 4.94%; NBQX: 13.6 ± 3.54%; Apocynin: 19.4 ± 4.14%). 

This data shows that combined AMPA receptor and NADPH oxidase inhibition results in a significant recovery in hind limb locomotor behavior following SCI. Apocynin was the only single treatment that was able to induce recovery in the BMS assessment, providing confirmatory data demonstrating that targeting oligodendrocyte preservation is important for the recovery of motor movement but alone is not sufficient to induce large changes in motor improvement. 

### Combined inhibition of AMPA receptors and NADPH oxidase improves tissue pathology following SCI

**Figure 5:**
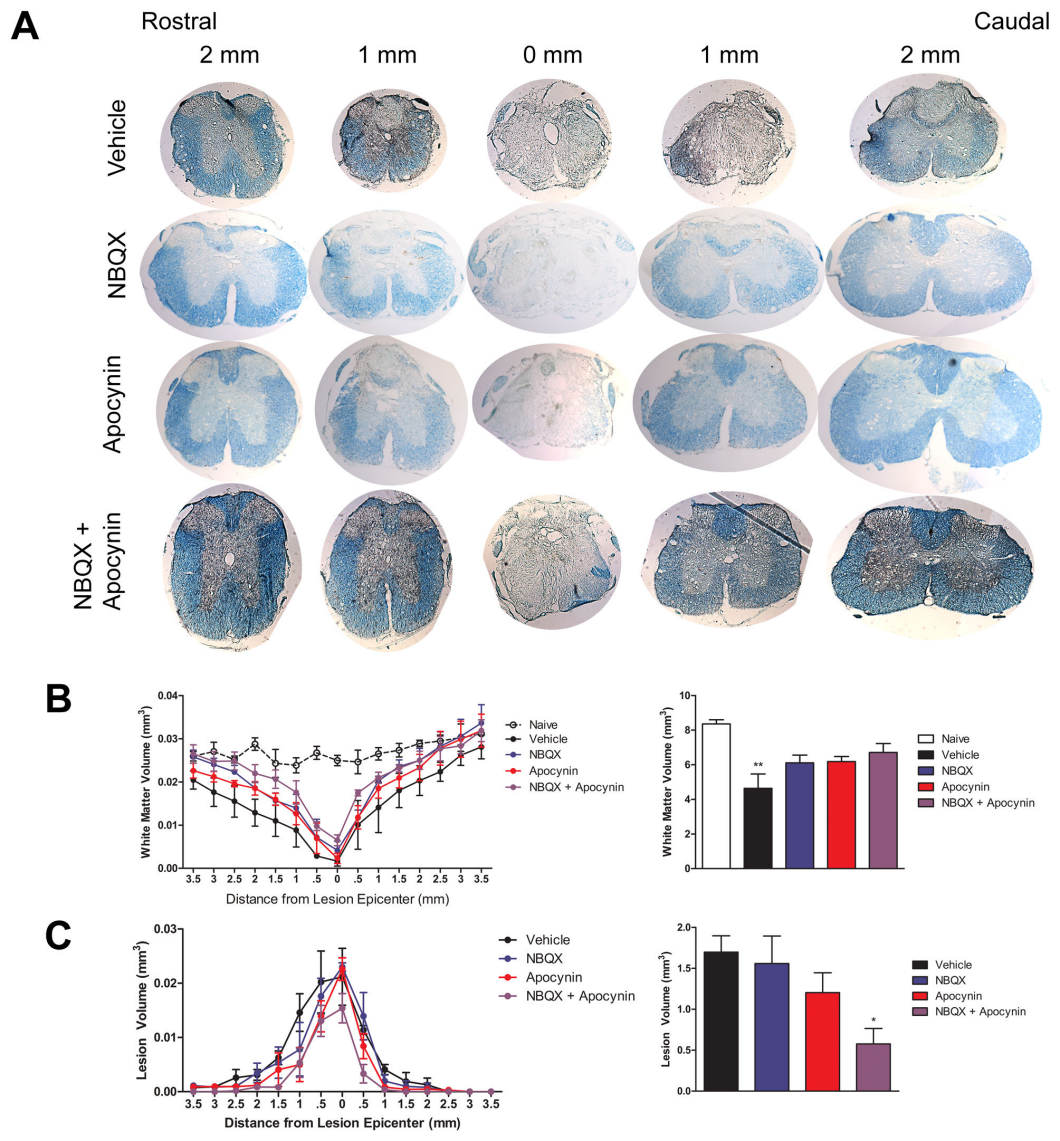
Combined treatment reduces white matter pathology and lesion volume following spinal cord injury. (A) Representative sections at 12 weeks post-injury for Luxol Fast Blue stained myelin from 2 mm rostral through 2 mm caudal of the lesion epicenter. Distribution (*left*) and quantification (*right*) of (B) intact myelin and (C) lesion volume 3.5 mm rostral to 3.5 mm caudal of lesion epicenter (n=3-4). *, p ≤ 0.05; **, p ≤ 0.01 compared to vehicle. Error bars indicate the standard error of the mean.

 To investigate whether AMPA and NADPH oxidase inhibition reduces white matter damage and lesion volume after SCI, spinal cords were stained for Luxol Fast Blue to visualize intact myelin and lesion size at 12 weeks after injury. Volume analysis revealed a significant reduction of intact myelin in vehicle treated animals (4.64 ± .83 mm^3^) when compared to uninjured controls (8.35 ± .24 mm^3^; Figure 5A, B). NBQX, apocynin, and combined treatment all preserved myelin after injury (6.11 ± .45, 6.18 ± .29, and 6.71 ± .51 mm^3^). Total spinal cord volume was not significantly changed between treatment groups, suggesting that the reduction in myelin volume was the result of myelin loss and not a reduction in spinal cord volume in the vehicle treated condition. Quantification of lesion volume revealed a significantly reduced lesion in combined treatment animals (.577 ± .19 mm^3^) compared to vehicle controls (1.7 ± .2 mm^3^; Figure 5C). NBQX or apocynin alone did not significantly affect total lesion volume (1.56 ± .34 and 1.20 ± .24 mm^3^), suggesting that any behavioral recovery following NBQX or apocynin treatment was probably due to the preservation of myelin and not a reduction in overall lesion size. Surprisingly, NBQX treated animals had a lesion volume comparable to vehicle treated controls, indicating, at least in this study, NBQX alone did not reduce necrotic damage. The combined treatment effectively reduced white matter damage and lesion volume, indicating that axon damage was potentially reduced. 

### Combined inhibition of AMPA receptors and NADPH oxidase reduces descending serotonergic fiber loss after SCI

**Figure 6:**
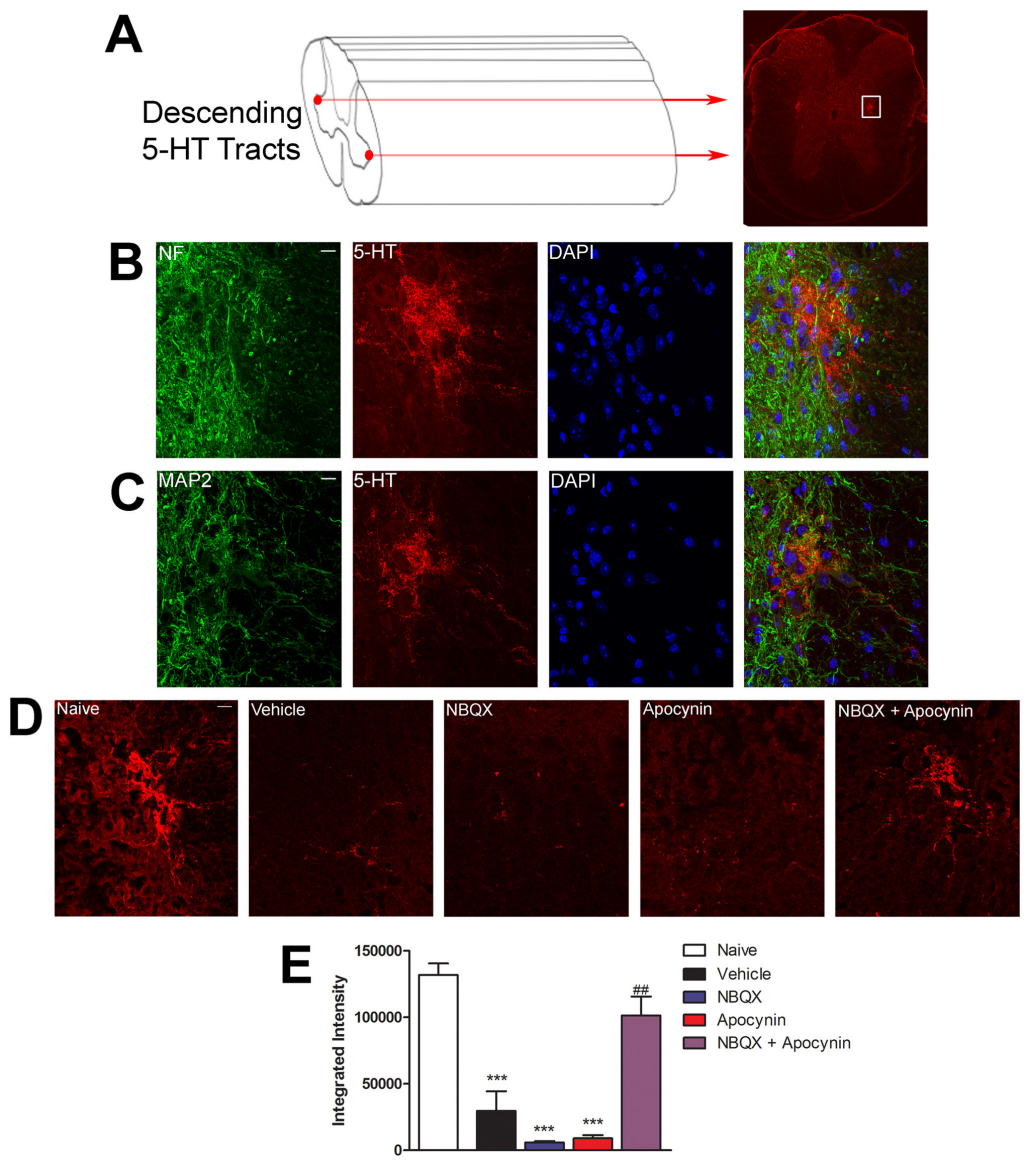
Combined inhibition of AMPA receptors and NADPH oxidase reduces descending serotonergic fiber loss after spinal cord injury. (A) Diagram and spinal cord cross-section showing the location of descending serotonergic tracts. White box represents the bilateral area used for quantification. (B,C) Co-labeling of serotonergic fibers in the spinal cord with neurofilament and MAP2. (D) Quantification of serotonergic immunofluorescence at 3 mm caudal to lesion epicenter revealed significantly more serotonin reactivity below the level of the lesion following treatment of NBQX + Apocynin (n = 3-4). (E) Representative magnified images from the white box in (A) depicting descending serotonergic tracts 3 mm caudal to lesion epicenter. Scale bar = 20 µm. ***, p ≤ 0.001 compared to naïve; ##, p ≤ 0.01 compared to vehicle. Error bars indicate the standard error of the mean.

Tract tracing studies have demonstrated the projection of serotonergic axons from the raphe nuclei in the brainstem throughout the spinal cord along the raphespinal tract adjacent to the gray matter lateral horn [18]. These descending serotonergic projections are important for fine motor control of coordinated movements, and preservation of these serotonergic projections has been correlated with the ability of mice to elicit controlled coordinated movements 2 months after SCI [18]. Since we observed significant recovery in motor behavior using two independent behavioral assessments with the combined therapy, we sought to evaluate the integrity of descending serotonergic tracts below the level of the lesion (Figure 6). Serotonin staining in naïve animals identified bilateral fiber bundles adjacent to the lateral horn of the gray matter which descended throughout the length of the spinal cord. This pattern of staining is similar to what has been demonstrated in the published literature [46]. Double-labeling of naïve mouse spinal cord sections showed these serotonergic fibers stained positive for the neuronal markers neurofilament and MAP2, although neurofilament does not predominantly label these fibers [47]. Quantification of relative serotonin reactivity of these bundles 3 mm caudal to the lesion epicenter showed a significant loss in immunoreactivity in vehicle, NBQX, and apocynin treated animals [Vehicle: 29,627 ± 14,844 Integrated Intensity (I.I.); NBQX: 5,993 ± 919.4 I.I.; Apocynin: 9,082 ± 2,364 I.I.]. Animals receiving combined treatment (101,434 ± 14,210 I.I.) had no significant loss is serotonin reactivity caudal to the lesion when compared to naïve (131,945 ± 8,557 I.I.) animals. The presence of serotonergic fibers below the level of the lesion in combined treated animals complements the behavioral assessments demonstrating the ability of the combined treatment to induce motor recovery and coordination.

## Discussion

In a previous study, we showed that injured astrocytes can influence oligodendrocyte survival through the regulation of extracellular glutamate and zinc levels [16]. We showed that media taken from injured WT astrocytes can elicit oligodendrocyte death in vitro, while media from injured GFAP-IκBα-dn astrocytes failed to elicit oligodendrocyte death. Further analysis demonstrated that in the presence of high glutamate levels, reduced extracellular zinc concentrations can potentiate oligodendrocyte death. This finding was found to be the result of the inability of zinc to reduce glutamate excitotoxicity through the attenuation of AMPA receptor activation. By maintaining basal extracellular zinc levels, either through inactivation of astroglial NF-κB or through zinc supplementation, oligodendrocytes were protected in the presence of high glutamate concentrations. 

In order to follow-up with these initial studies, we investigated the oxidative stress response in WT and GFAP-IκBα-dn animals following a moderate contusion SCI. Peak expression of gp91 phox, the catalytic subunit of the NADPH oxidase enzyme, following SCI occurs at 1 week after injury and coincides with the second wave of apoptotic oligodendrocyte death [48-50]. In order to determine if ROS could be implicated in glutamate/zinc-mediated oligodendrocyte death, we wanted to determine if there were differences in the amount of oxidative damage between WT and GFAP-IκBα-dn mice at 1 week following SCI. By measuring changes in protein oxidation, we were able to show that WT animals had significantly more oxidative damage, and these changes correlated with higher gp91 phox expression.

Despite possessing significantly lower levels of gp91 phox protein expression than WT mice, SCI resulted in a several fold increase in the amount of gp91 phox in GFAP-IκBα-dn animals. The reason for this increase in unclear, however, the expression of gp91 phox is not limited to astrocytes. Microglia, neurons, endothelial cells, fibroblasts, and macrophages all express NADPH oxidase as well. It is possible that the increase in gp91 phox observed in the GFAP-IκBα-dn group is a compensatory mechanism influenced by these additional cell types, or it could be that the astrocytes were able to increase the expression of gp91 phox through an alternative signaling pathway not involving NF-κB. Additional studies would need to be performed in order to elucidate these complex cellular interactions.

Additional studies have shown that high levels of extracellular glutamate induce excitotoxic cell death involving the production of ROS [5,51]. Since we demonstrated the inactivation of NF-κB in astrocytes can reduce the oxidative stress response after injury, we wanted to determine if injured astrocyte media lacking sufficient levels of zinc to prevent oligodendrocyte toxicity would induce activation of the NADPH oxidase enzyme. We found that treatment of oligodendrocytes with injured astrocyte media with a reduction in zinc levels was sufficient to result in NADPH oxidase activation. Furthermore, treatment of gp91 phox-deficient oligodendrocytes with excitotoxic levels of glutamate and varying levels of zinc showed that oligodendrocytes lacking functional NADPH oxidase are protected from glutamate excitotoxicity. These findings suggest that after trauma astrocytes can regulate oligodendrocyte death by reducing extracellular zinc concentrations and initiating NADPH oxidase activation within the oligodendrocyte through stimulation of Ca^2+^-permeable AMPA receptors. 

Excitotoxicity in neurons stimulates a rapid production of superoxide resulting in cell death [5]. Brennan et al. [5] were able to block these events by inhibiting NADPH oxidase activity. Indeed, by pre-treating oligodendrocytes with an NADPH oxidase inhibitor, apocynin, oligodendrocyte toxicity was reduced to control levels. We were able to confirm these results by treating *Cybb* ^*-/-*^ oligodendrocytes devoid of gp91 phox with injured astrocyte media. Oligodendrocytes lacking gp91 phox were protected from injured astrocyte-mediated cell death. 

These *in vitro* results were verified *in vivo* following a moderate contusion SCI. Daily administration of 10 mg/kg apocynin was able to reduce oligodendrocyte death by 75% at 1 week following SCI. Experiments using a vascular clamp model of spinal cord injury showed that administration of a 5 mg/kg apocynin at 1 and 6 hrs after injury completely blocked cell death within the spinal cord and induced a significant increase in locomotor behavior by 4 days after injury [31]. Using a contusive spinal cord injury model, our data suggests that while daily administration of 10 mg/kg apocynin was highly effective at reducing oligodendrocyte death, this change was only able to produce a modest improvement in behavioral outcome, possibly due to the inability of NADPH oxidase inhibition to block axonal damage (Figure 6). The discrepancy in apocynin potency between Impellizzeri et al. [31] and our work shown here can likely be attributed to the differences in injury model and injury severity. Dose ranging studies with different injury severity need to be performed in order to elucidate the potential of apocynin as a therapeutic treatment. 

Contrary to the NF-κB transcription factor, inhibition of the NADPH oxidase enzyme alone did not result in drastic improvements in functional recovery. The reason for this result is unclear, but NF-κB regulates a variety of proteins involved in the inflammatory response which could be detrimental to axon function [17]. Interestingly, glutamate excitotoxicity has been implicated in axon dysfunction as well. Mice lacking myelin basic protein (MBP), a protein necessary for proper myelin formation and compaction, were treated with either AMPA or N-methyl-D-aspartate (NMDA) in order to determine the role of AMPA and NMDA receptors in axonal dysfunction [41]. Pitt and colleagues found that axons deficient in proper myelination were susceptible to AMPA over-excitation, indicating that spinal axons are to excitotoxicity. These findings were supported by previous experiments by Tekkok et al. [42] demonstrating that AMPA receptor blockade inhibited axon dysfunction and white matter pathology. NMDA antagonism had not effect in this model. 

Recognizing that oligodendrocyte preservation alone is likely insufficient to promote functional recovery after contusive SCI, we employed a combinatorial treatment strategy to reduce oligodendrocyte death and block axonal damage. Axonal AMPA receptor activation has been identified as a mediator of axon damage [41,42], and the AMPA receptor antagonist NBQX has previously been used in models of spinal cord injury and neurological disease [43-45]. Combined treatment with NBQX and apocynin resulted in a significant improvement in hind limb motor behavior as measured by BMS and grid walk analysis. Histological analysis showed that NBQX, apocynin, and combined treatment all significantly preserved white matter after injury, while the combined therapy was the only treatment that reduced lesion volume. Surprisingly, NBQX treatment did not reduce lesion volume as previously shown [43-45]. Apocynin alone failed to reduce total lesion volume, thereby providing confirmatory data demonstrating that white matter preservation alone is not sufficient to regain complete functional recovery. 

Contrary to the individual treatments, combined treatment of apocynin and NBQX significantly reduced lesion size, in addition to preserving white matter, potentially due to an additive effect of AMPA receptor and NADPH oxidase inactivation in reducing free radical production and axonal damage as a result of calpain inhibition [51]. In addition, it is possible that the AMPA receptor and NADPH oxidase activation pathways are similar, but the differences in their signaling pathways may be sufficient enough to confer added production when targeted together. 

Of particular note is the preservation of serotonergic fibers below the level of the lesion by the combined treatment of NBQX and apocynin. Serotonin has previously been linked to the regulation of patterned walking in adult animals [30,52] and its presence could account for the improvement in locomotor behavior as measured by two independent behavioral assessments. As a result, combined inhibition of AMPA receptors and NADPH oxidase appear to be viable therapeutic targets to induce functional recovery following SCI.

This concept of targeting two proteins in similar signaling pathways has previously been established as vertical inhibition. Previous work performed by Werzoma et al. [53] showed that using vertical inhibition treatment strategies can be effective in melanoma research. Werzoma and colleagues were able to show that either targeting phosphoinositide 3-kinase (PI-3K) or mammalian target of rapamycin (mTOR) was sufficient to induce melanoma cell apoptosis, but when dual targeted these inhibitors resulted in a synergistic suppression of cellular proliferation. Using inhibitors to block two different proteins in the same signaling cascade, Werzoma and colleagues [53] were able to induce superior protection against melanomas *in vitro* and *in vivo*. This form of combinatorial strategy could lead to novel therapeutics in the field of medicinal research. 

These studies demonstrate that oligodendrocytes express NADPH oxidase, and activation of this enzyme is triggered by injured astrocytes. Pharmacological and genetic inhibition of NADPH oxidase rendered oligodendrocytes immune to the cytotoxic effects of injured astrocytes. Combinatorial strategies inhibiting NADPH oxidase and the AMPA receptor resulted in significant improvements in functional recovery, reduced white matter damage, and improved axon sparing following SCI. These studies provide a novel understanding of the ability of astrocytes to regulate oligodendrocyte fate following SCI, and provide valuable insight into previously unknown mechanisms governing oligodendrocyte death following trauma.
